# Extremely wideband ridge gap waveguide-based 3-dB coupler using supershaped coupling apertures

**DOI:** 10.1038/s41598-025-23099-4

**Published:** 2025-11-10

**Authors:** Davood Zarifi, Ali Sabbaghi Saber, Ali Farahbakhsh, Michal Mrozowski

**Affiliations:** 1 Department of Microwave and Antenna Engineering, Faculty of Electronics, Telecommunications, and Informatics, Gdańsk University of Technology, 80-230 Gdańsk, Poland; 2 School of Electrical and Computer Engineering, University of Kashan, Kashan, Iran; 3 Department of Electrical and Computer Engineering, Graduate University of Advanced Technology, Kerman, Iran

**Keywords:** Engineering, Optics and photonics, Physics

## Abstract

This paper introduces a novel broadband 3-dB coupler based on ridge gap waveguide (RGW) technology designed using supershapes to achieve exceptional bandwidth while maintaining a compact footprint. By leveraging the superformula—a parametric model capable of generating complex geometries with minimal computational effort-the proposed design enables efficient exploration of a wide design space. Furthermore, to ensure efficient excitation of the coupler across the entire operational bandwidth two novel transitions from RGW to coaxial line and double-ridge waveguide are designed. The proposed coupler achieves a fractional bandwidth of 75.8% over the 18–40 GHz range (covering both K and Ka bands), featuring a return loss better than 18 dB, a coupling level of 3 ± 0.3 dB, isolation exceeding 22 dB, and a significantly compact footprint of 0.8λg—substantially smaller than conventional designs. Simulation and measurement results confirm the effectiveness of the proposed approach, demonstrating its suitability for high-performance broadband applications in microwave and millimeter-wave (mmWave) systems.

## Introduction

Couplers play a crucial role in radio frequency (RF) and microwave systems, facilitating efficient signal distribution and isolation in various applications. These components are widely used in telecommunications, test and measurement setups, and radar systems, where they enable power monitoring, signal splitting, and impedance matching^1^. By allowing controlled signal coupling between transmission lines, directional couplers enhance system performance, minimize interference, and support accurate signal analysis. Wideband couplers can be implemented using various technologies, including planar printed circuit board (PCB) structures and hollow waveguides, each offering distinct advantages. PCB-based couplers provide a compact and cost-effective solution, making them ideal for integration into modern RF systems^2^. However, their performance is constrained by dielectric losses and limited power handling, particularly at higher frequencies. In contrast, hollow waveguide couplers excel in high-power applications where minimal insertion loss and superior isolation are required, making them well-suited for mmWave and aerospace systems.

Wideband hollow waveguide-based couplers are commonly designed using two closely spaced parallel waveguides connected through a series of apertures in either the broad or narrow walls. Multi-section configurations are advantageous for achieving broad bandwidth but require longer structures due to multiple apertures and spacing requirements^3^. For example, a compact 3-dB E-plane waveguide directional coupler has been developed using large apertures in the broad walls of two parallel waveguides, resulting in high directivity and stable coupling performance^4^. Another variation, operating in Ku-band, demonstrated excellent efficiency^5^. Additionally, 3-dB directional couplers can be realized using branch-line waveguide structures, where multiple branch lines connect adjacent waveguides^6–9^. These designs enable broad frequency operation with stable coupling characteristics but these designs result in narrow branch lines which may limit power handling and structural compactness.

Hollow waveguide-based couplers face challenges at higher frequencies due to the precision and cost associated with their manufacturing, particularly in achieving reliable electrical connections between layers. To address these limitations, gap waveguide technology has emerged as a viable alternative to hollow waveguides, enabling the development of various high-frequency components^10^. For instance, several 3-dB directional couplers offering solutions with varying bandwidths and phase balance characteristics, based on printed ridge gap waveguide (PRGW) have been explored^11–15^. While PRGWs are cost-effective due to PCB fabrication, they are not suitable for high-power applications. To address power-handling limitations, researchers have introduced full metal gap waveguide-based 3-dB couplers, providing improved insertion loss and isolation^16–22^. For instance, a compact ridge gap waveguide (RGW) hybrid coupler proposed in^16^ demonstrated low insertion loss but had a relatively narrow bandwidth of 14%. Another study in^19^ presented a broadband 3-dB directional coupler using a groove gap waveguide (GGW), operating from 57 to 74 GHz with a bandwidth of 26%. Furthermore, a gap waveguide-based 3-dB branch-line coupler operating across 13 to 22 GHz has been proposed in^21^; however, its large size is a significant limitation for compact system integration. More recently, study^22^ has reported the design of a compact, wideband 3-dB directional coupler based on a cruciform structure, achieving coupling levels of 3 ± 0.5 dB at the output ports across the 17.9–24 GHz frequency range. Despite recent advancements in gap waveguide-based couplers with enhanced power-handling and bandwidth capabilities, achieving optimal broadband impedance matching while maintaining a compact form factor remain a significant challenge.

This study presents a novel approach to designing a highly efficient, broadband and at the same time compact RGW-based coupler by employing supershapes. The key innovation of this methodology is the use of parametric geometries in the design to maximize bandwidth while maintaining a minimal footprint and ensuring compatibility with standard fabrication techniques. The advantage of employing supershapes in coupler design lies in their ability to precisely define intricate structural profiles using a small set of tunable parameters, enabling an efficient exploration of a vast design space in an optimization process. Without this approach, identifying the structural configuration that meets the requirements for compactness, broadband operation, and good electromagnetic performance would be extremely complex and computationally expensive, even with state-of-the-art optimization algorithms.

The frequency band of interest for the coupler is from 18 to 40 GHz. This frequency band is essential to modern communication and radar technologies, providing high data transmission speeds and enhanced resolution across numerous applications. This spectrum is extensively employed in satellite communications, 5G networks, and military radar systems, where its capacity for handling high-bandwidth signals ensures superior connectivity and precision. To demonstrate the effectiveness of the proposed approach, we introduce the design of a parametrically optimized RGW-based 3-dB coupler featuring an exceptional fractional bandwidth of 75.8% within the 18–40 GHz range. This remarkable broadband performance is achieved despite a significantly smaller footprint compared to previously reported gap waveguide-based couplers.

The coupler design is complemented by the development of two new broadband transitions: one from a ridge gap waveguide to a coaxial line using a standard 2.92 mm coaxial connector, and another from a ridge gap waveguide to a standard WDR-180 double-ridge waveguide. It is worth noting that several broadband transitions from ridge gap waveguides to coaxial lines have been proposed in the literature, typically involving complex and relatively large structures^23–25^. The predominant strategy in these designs involves first creating a transition from the coaxial line to either a single- or double-ridge waveguide, followed by an adapter section that converts the ridge waveguide mode to the RGW mode. The only known exception is the design presented in^25^, where the RGW is directly fed from a coaxial line. However, that design supports operation only up to 37 GHz, falling short of the 40 GHz upper limit of a standard 2.92 mm coaxial connector and also the upper limit of the Ka band. This bandwidth was defined based on achieving a reflection loss better than 15 dB. The proposed coupler is designed to operate across the full K and Ka bands (18–40 GHz). Therefore, a new transition is required to directly interface a standard 2.92 mm coaxial connector with the RGW preferably with even better matching than the previously reported designs. Additionally, to enable integration with a waveguide flange, which is needed for high-power operation, we must develop a transition from the standard WRD-180 double-ridge waveguide, which also operates from 18 to 40 GHz, to the RGW. To the best of the authors’ knowledge, such a transition has not yet been reported in the literature Therefore, in addition to a compact broadband coupled this work introduces also a novel transition to address this gap.

## Design of coupler

### Gap waveguide technology

**Fig. 1:**
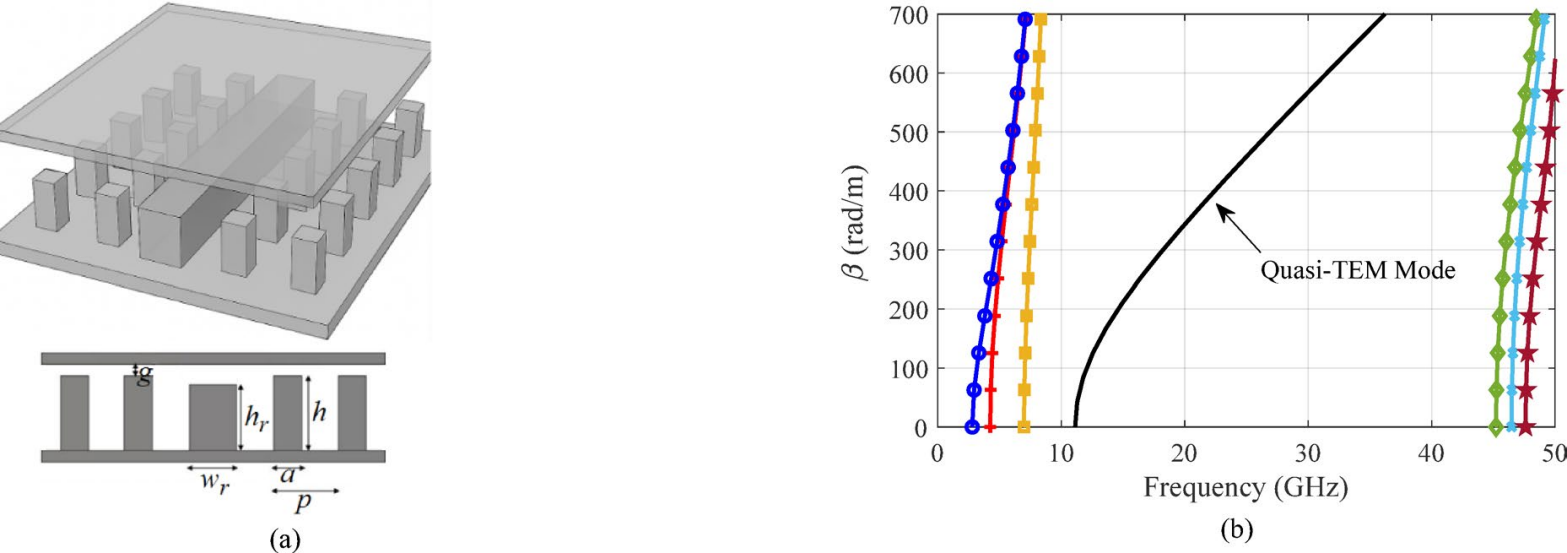
(**a**) Unitcell of periodic pin structure. (**b**) Dispersion diagram for the first three modes.

Gap waveguide technology utilizes a parallel-plate waveguide to control electromagnetic wave propagation. The wave is guided within a channel formed between two electrically isolated metal layers, with lateral confinement provided by periodic arrays of metallic pins. The geometry of RGW structure is illustrated in Fig. 1a. By adjusting the geometric parameters, the desired stop-band can be achieved^10^. For achieving a stop-band covering 18–40 GHz, the geometric parameters are *g* = 0.05 mm, *a* = 1.5 mm, *p* = 4 mm, *h* = 3 mm, *w*_*r*_ =3 mm and *h*_*r*_ =1.87 mm. The dispersion characteristics of the RGW structure are depicted in Fig. 1b.

**Fig. 2:**
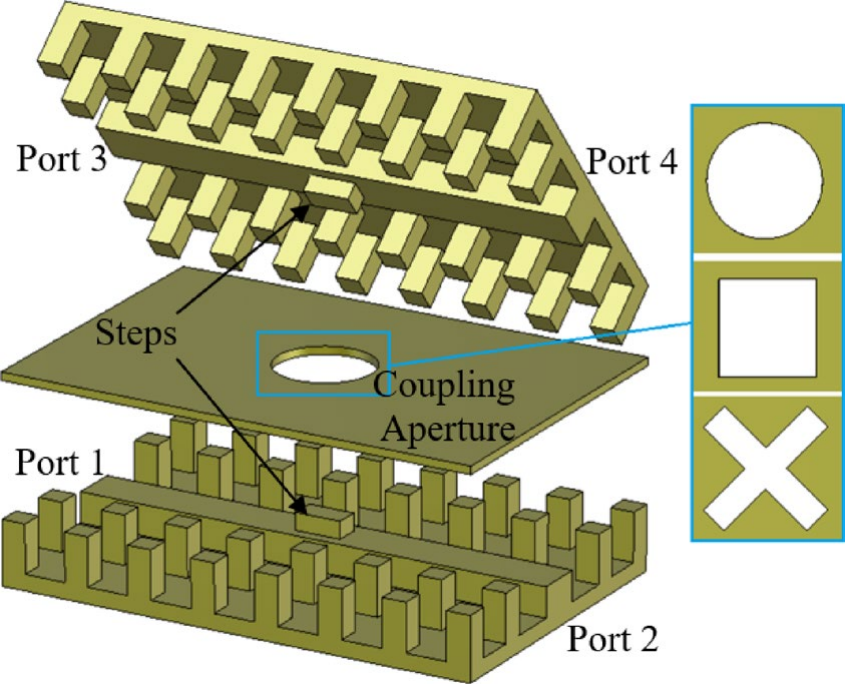
Exploded view of coupled parallel ridge gap waveguides.

### Coupled parallel RGWs

To achieve an exceptionally wide single-mode bandwidth spanning the 18–40 GHz range, RGWs can be employed as waveguide channels. These waveguides provide broader bandwidth and a more compact design compared to groove gap waveguides^10^. As illustrated in Fig. 2, waveguide coupling can be achieved through an aperture, with various hole geometries-including circular, rectangular, and cross-shaped-previously explored in the literature^26^. To enhance matching and regulate the level of coupling, a step (*l*_*s*_×*w*_*s*_×*h*_*s*_) is introduced in each ridge adjacent to the coupling aperture. Ports 1, 2, 3, and 4 are the input, through, coupled, and isolated ports, respectively. The coupling coefficient (C), directivity (D), and isolation (I) are defined as follows:

1 $$C\,=\,\,10\,\log \,\left| {\frac{{{P_{in}}}}{{{P_f}}}} \right|=\,\,20\,\log \,\left| {\frac{1}{{{S_{31}}}}} \right|$$

2 $$D\,=\,\,10\,\log \,\left| {\frac{{{P_f}}}{{{P_b}}}} \right|\,=\,\,20\,\log \,\left| {\frac{{{S_{31}}}}{{{S_{41}}}}} \right|$$

**Fig. 3:**
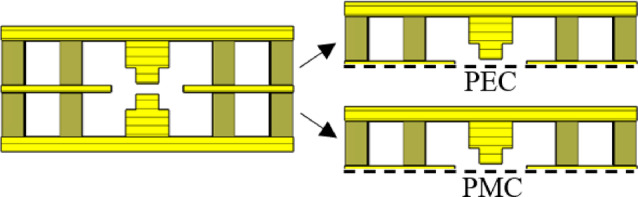
Central cross-section of the coupler and even and odd modes. The step dimensions on the ridges are defined by a width *w*_*s*_ = 1.54 mm and a height *h*_*s*_ = 1.07 mm, while the coupling region between the waveguides spans a width of 5 mm.

**Fig. 4:**
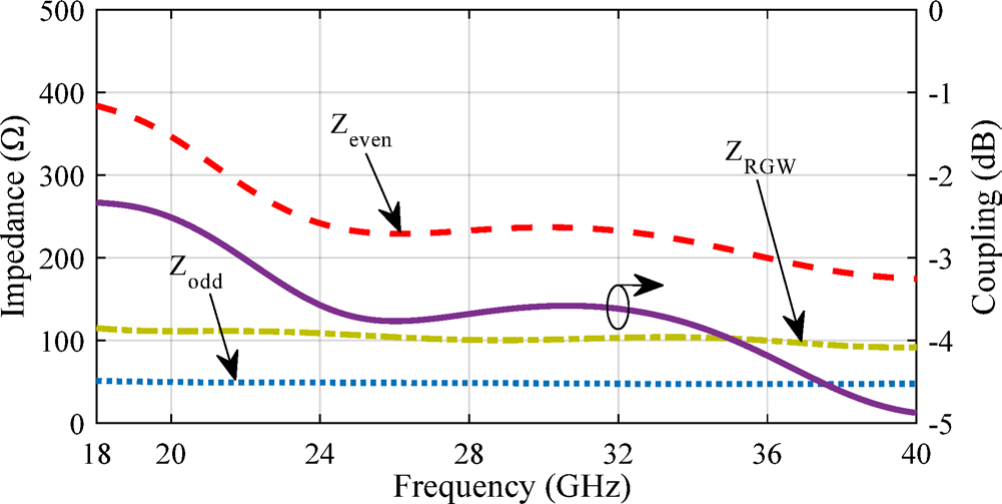
Impedance values for odd and even modes.

3 $$I\,=\,\,10\,\log \,\left| {\frac{{{P_{in}}}}{{{P_b}}}} \right|\,\,=\,\,20\,\log \,\left| {\frac{1}{{{S_{41}}}}} \right|\,=\,\,C+\,\,D$$

where *P*_*in*_, *P*_*f*_ and *P*_*b*_ are the incident power at the input port, the coupled power, and the power output of the isolated port, respectively.

To analyze the coupling between the waveguide channels, the well-established model based on even and odd impedances can be utilized^27^. As shown in Fig. 3, the central cross-section of the structure features a relatively large coupling aperture. By imposing Perfect Magnetic Conductor (PMC) or Perfect Electric Conductor (PEC) boundary conditions at the symmetry plane and solving the corresponding two-dimensional eigenvalue problems, the even and odd modes can be systematically characterized. Numerical techniques in CST Microwave Studio are employed to extract the characteristic impedances of the even and odd modes within this cross-section. These impedances, along with the characteristic impedance of the RGW, are depicted in Fig. 4. Furthermore, the coupling value (in dB) is determined using

4 $$C\,=\,20\,\log \,\left| {\frac{{{Z_{even}} - {Z_{odd}}}}{{{Z_{even}}+{Z_{odd}}}}} \right|.$$

and plotted. As illustrated in Fig. 4, the coupling between the parallel waveguides varies approximately between 2 dB and 5 dB across the 18–40 GHz frequency band. This behavior is significant from two main perspectives. First, the observed fluctuations in coupling coefficients underscore the sensitivity of the structure to geometric parameters. Achieving a stable coupling level of approximately 3-dB over a wide frequency range requires deliberate modifications to the design—particularly in the width of the coupling region and the height and width of the steps on the ridges. Second, the dimensional values selected for the coupler hole and ridge steps in this initial analysis provide a practical foundation for subsequent optimization. These parameters will be further refined using the supershape approach, as discussed in the following section. The primary objective is to identify an aperture geometry and step dimension that consistently achieve 3-dB coupling across the entire operational bandwidth of 18 to 40 GHz.

**Fig. 5:**
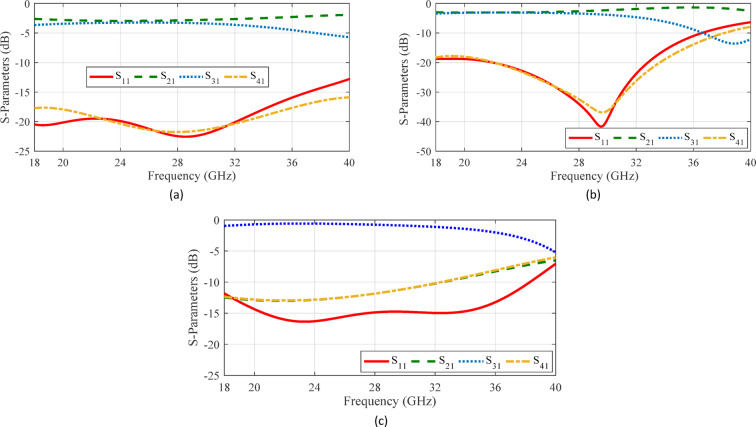
Simulated S-parameters of coupled parallel RGWs with different coupling apertures. (**a**) Circular shape with a radius of 2.45 mm, *l*_*s*_ = 2.70 mm, *w*_*s*_ = 1.58 mm and *h*_*s*_ = 1.05 mm. (**b**) Square shape with a side of 4.50 mm, *l*_*s*_ = 2.90 mm, *w*_*s*_ = 1.38 mm and *h*_*s*_ = 1.02 mm. (**c**) Cross shape with two 14.4 mm × 2.4 mm slots, *l*_*s*_ = 3.04 mm, *w*_*s*_ = 2.99 mm and *h*_*s*_ = 1.12 mm.

As an initial approach to meeting the desired performance criteria, the coupler structure is optimized using three simple aperture shapes: circular, square, and cross-shaped. Figure 5 illustrates the simulation results for two parallel coupled RGWs connected via simple coupling apertures. As can be seen, the shape of the aperture significantly influences the coupler’s bandwidth and these conventional shapes fails to meet the desired specifications of the coupler—such as excellent input port matching and balanced output power distribution—across the entire 8–18 GHz frequency range. Consequently, selecting more complex hole geometry is expected to improve the bandwidth and impedance matching.

**Fig. 6:**
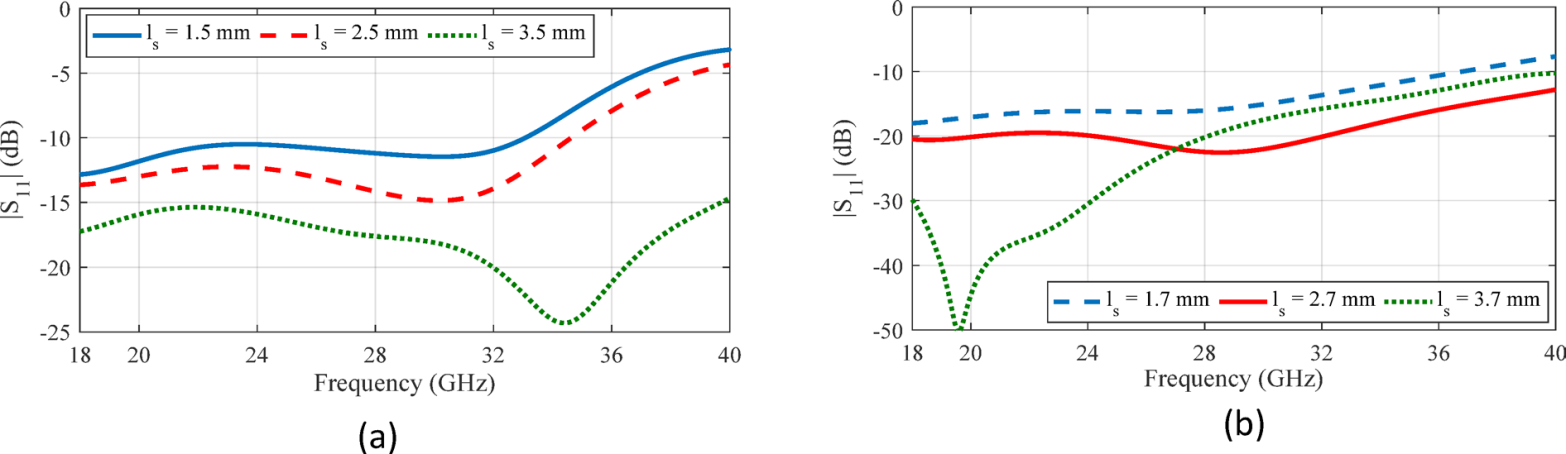
The impact of steps created on the ridges on the performance of parallel ridge gap waveguides with circular coupling aperture.

To evaluate the impact of steps formed on the ridges on the coupler’s performance, parametric sweeps for the step’s height and length are conducted, with the simulation results illustrated in Fig. 6. These results demonstrate that the step dimensions substantially affect the bandwidth, indicating a strong sensitivity of the coupler’s electromagnetic behavior to even minor geometric variations. Therefore, to achieve optimal performance, the step geometry must be carefully optimized in conjunction with the aperture shape. A holistic design approach that simultaneously considers both features enables a more robust and broadband coupler configuration.

### Supershaped aperture coupler

The superformula, originally formulated by Johan Gielis, presents a generalized mathematical approach to describing intricate geometrical shapes using a compact set of parameters^28^. A wide range of complex curves and patterns can be derived as extensions of basic circular forms. Defined within the framework of polar coordinates as.

**Fig. 7:**
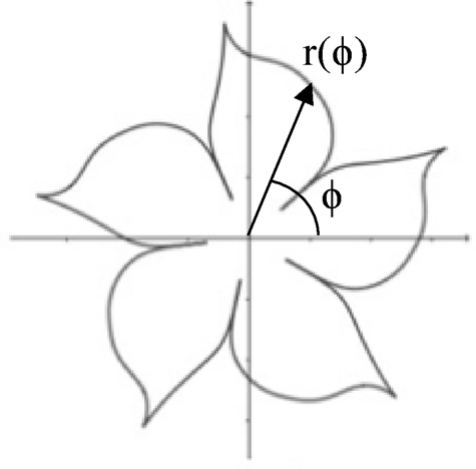
Typical supershape generated by superformula.

**Fig. 8:**
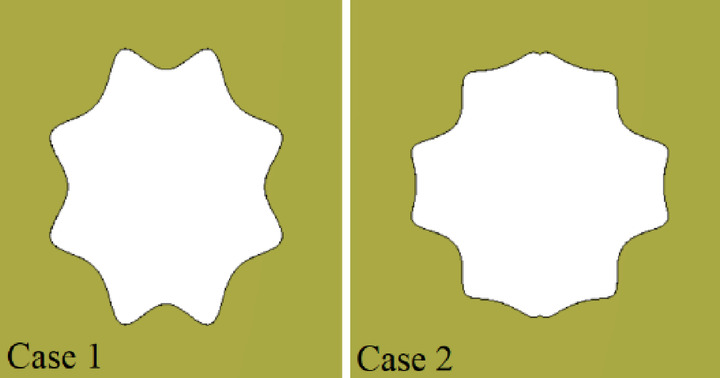
Two typical supershaped coupling apertures.

5 $$r(\varphi )=\,{\left[ {{{\left| {\frac{1}{{{r_0}}}\cos \left( {\frac{m}{4}\varphi } \right)} \right|}^{{n_2}}}+\,\,{{\left| {\frac{1}{{{r_1}}}\sin \left( {\frac{m}{4}\varphi } \right)} \right|}^{{n_3}}}} \right]^{{{ - 1\,} \mathord{\left/ {\vphantom {{ - 1\,} {\,{n_1}}}} \right. \kern-0pt} {\,{n_1}}}}}$$

where radius and angle are key variables, the equation provides a versatile tool for modeling natural and abstract structures by varying six arbitrary numerical parameters *m*, *n*_1_, *n*_2_, *n*_3_, *r*_0_, and *r*_1_. A typical supershape generated by superformula is illustrated in Fig. 7.

The efficiency of generating both intricate and straightforward curves with a minimal set of six parameters makes the superformula particularly valuable for computer-aided design, especially in shape optimization and electromagnetic analysis. As a result, recent advancements have leveraged this mathematical tool in the antenna and microwave engineering, including antennas^29–35^, frequency selective surfaces^36,37^, microstrip ring-hybrid couplers^38^, waveguide adaptors^39^ and twists^40^. In this study, supershapes are utilized to refine the geometry of the coupling hole of in a highly compact and wideband gap waveguide-based 3-dB coupler for mmWave applications.

The proposed 3-dB coupler is composed of two parallel RGWs coupled via a supershaped aperture. This design choice stems from the observations discussed in previous section, which demonstrated that conventional geometries-such as circular, rectangular, or cross-shaped apertures-are insufficient for achieving enhanced bandwidth. By describing the aperture shape using the superformula, a single equation can generate both traditional and highly complex geometries using only six parameters. This enables the creation of a wide variety of intricate shapes with remarkable flexibility, allowing for efficient optimization to identify the most suitable aperture for a given bandwidth. This approach significantly reduces computational complexity and accelerates the simulation process, as traditional shape-definition methods typically require a much larger number of variables to achieve comparable geometrical forms.

**Fig. 9:**
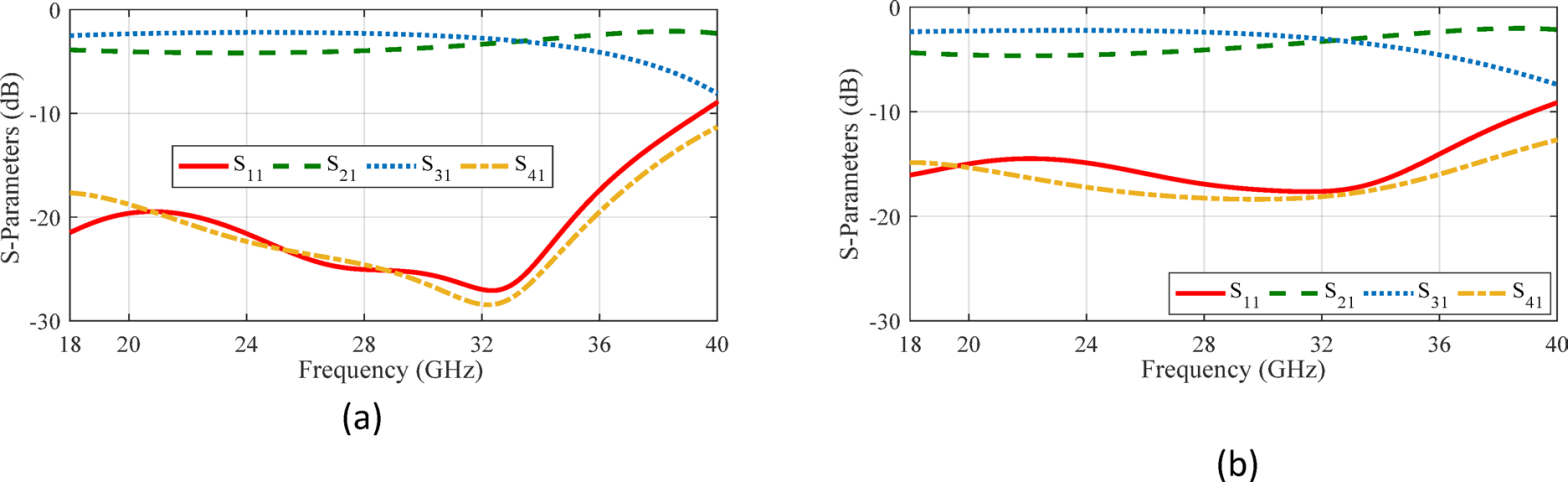
Simulated S-parameters of two typical couplers with supershaped coupling apertures. (**a**) Case 1. (**b**) Case 2.

To illustrate the impact of aperture shape on the coupler’s bandwidth, Fig. 8 shows two representative supershaped coupling apertures. The process of generating these shapes begins by creating a supercurve for each angular increment (*φ*) from 0 to *π*/2. This curve is then mirrored around the *x* and *y* axes to form a closed contour, ultimately resulting in a supershape. When the parameters *r*_0_ and *r*_1_ are left unrestricted, the radial value *r*(*φ*) can become extremely large at certain angles and very small at others. This variation often produces distorted geometries and causes simulation errors in CST. To mitigate this issue, we fixed these two parameters at 1 and applied directional scaling to control the overall dimensions of the shape. In this study, we introduced two independent scaling parameters (*s*_*x*_ and *s*_*y*_) for the *x* and *y* directions, respectively. The parameters used in superformula (1) to define these two apertures are as follows: Case 1: (*m* = 8, *n*_1_ = 4.40, *n*_2_ = 6.00, *n*_3_ = 4.00, *s*_*x*_ = 2.26 and *s*_*y*_ = 2.68), Case 2 (*m* = 10, *n*_1_ = 10, *n*_2_ = 4.00, *n*_3_ = 8.00, *s*_*x*_ = 3 and *s*_*y*_ = 3). These configurations have not been completely optimized; they are included solely to demonstrate the influence of design parameters. The corresponding reflection coefficients for these supershaped RGW couplers with matching steps on the ridges are presented in Fig. 9. A clear improvement in bandwidth compared to conventional apertures is observed.

**Fig. 10:**
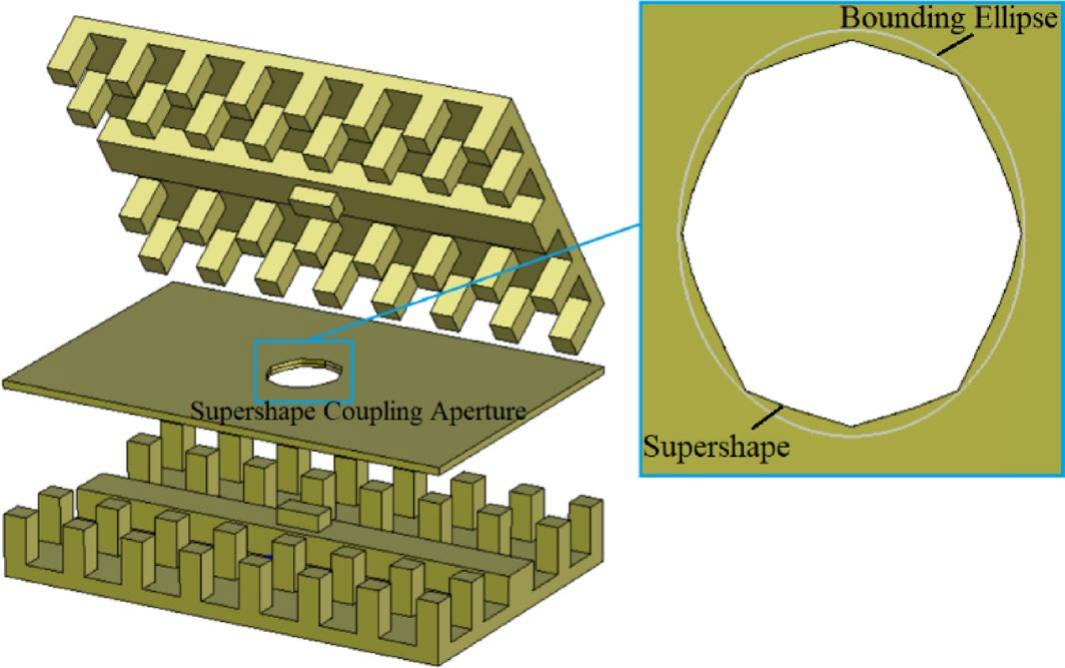
Configuration of optimized 3-dB coupler with supershaped coupling aperture.

## Optimization process

Fine-tuning the unknown variables in the proposed coupler design enables achieving the target design goals for input matching, coupling, and isolation performance. Ultimately, the geometrical parameters, including *m*, *n*_1_, *n*_2_, *n*_3_, and *s*_*x*_, *s*_*y*_ along with the step height (*h*_*s*_), and length (*l*_*s*_) and width (*w*_*s*_) on the ridges, are determined through an optimization procedure. The permissible range for these parameters is set as follows: *m*(2–10, integer values only), *n*_1_(1–10), *n*_2_(1-100), *n*_3_(1-100), *s*_*x, y*_(0.1-3), *h*_*s*_(0.1 –1.1 mm), *l*_*s*_(0.1 –3 mm) and *w*_*s*_(0.1 –2 mm). The optimization workflow utilizes the Trust Region algorithm within CST Microwave Studio. The goal is to develop a coupler that maintains an input matching better than − 20 dB, a flat coupling value of 3 ± 0.5 dB and isolation higher than 20 dB over the extensive frequency spectrum spanning 18 to 40 GHz.

**Fig. 11:**
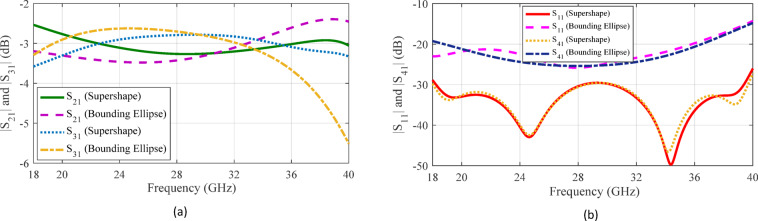
Simulated *S*-parameters of the optimized 3-dB coupler with supershaped coupling aperture.

**Fig. 12:**
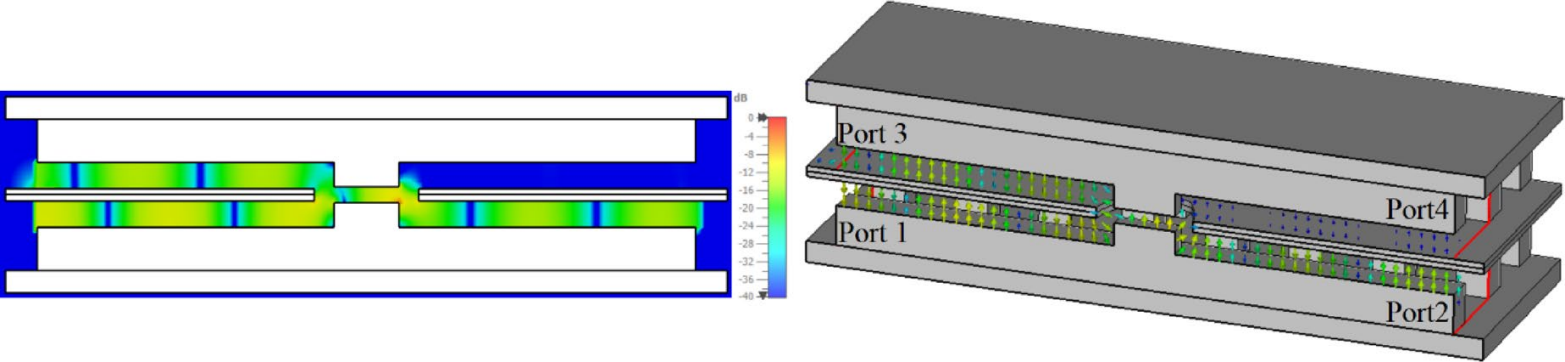
Simulated electric field distribution of 3-dB coupler at 29 GHz.

The supershaped gap waveguide coupler with optimized values (7, 4.40, 1.01, 1.34, 2.26, 2.69, 1.08 mm, 2.86 mm, 1.54 mm) for (*m*, *n*_1_, *n*_2_, *n*_3_, *s*_*x*_, *s*_*y*_, *h*_*s*_, *l*_*s*,_ *w*_*s*_) is displayed in Fig. 10. The optimal shape of the coupling aperture resembles an irregular octagon with unequal sides and rounded corners. To further examine the influence of the supershape on a basic geometry like an ellipse, a bounding elliptical shape is considered, as illustrated in Fig. 10. The *S*-parameters of the structures are illustrated in Fig. 11. Note that the for supershaped coupler, the reflection coefficient at the input port is below − 25 dB, indicating excellent input matching, while the isolation between ports 1 and 4 exceeds 25 dB. The transmission coefficients to ports 2 and 3 maintain a stable − 3 ± 0.5 dB across the entire bandwidth from 18 GHz to 40 GHz. In the case of the coupler with elliptical coupling aperture, the input port return loss and output port isolation exceed 15 dB, while the transmission coefficients to ports 2 and 3 remain within − 4 ± 1.5 dB throughout the entire operating bandwidth. A comparison of the simulation results for couplers with supershaped and elliptical coupling apertures demonstrates that the supershape-based design offers markedly better input port matching, superior isolation, and lower output port imbalance. The electric field distribution of the supershaped coupler at 26 GHz, as depicted in Fig. 12, illustrates the operation of the coupler and confirms the accuracy of the *S*-parameter results.

## Sensitivity analysis

**Fig. 13:**
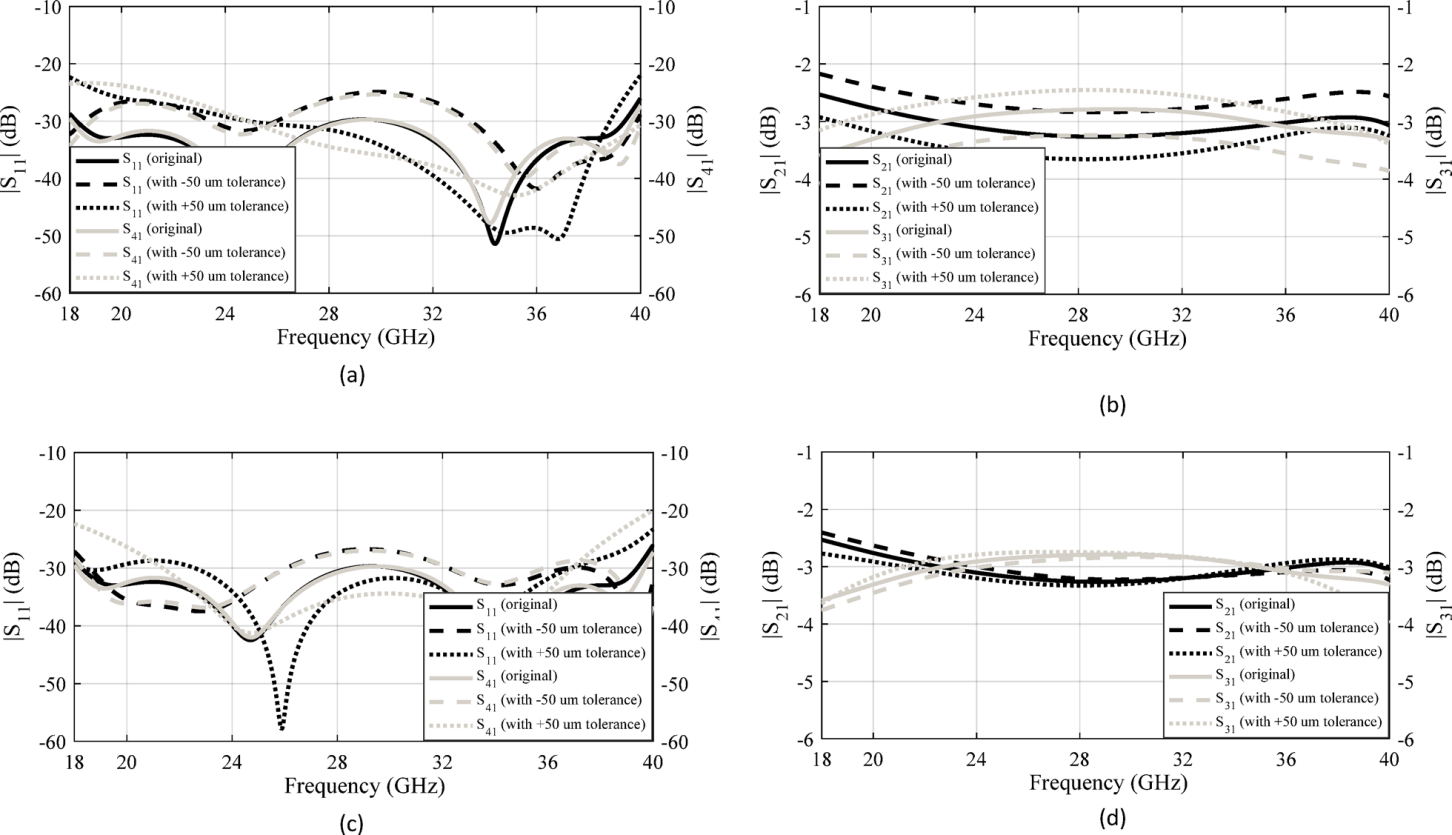
Simulated S-parameters of the 3-dB coupler considering a dimensional tolerance of ±50 μm: (**a**) and (**b**) variations in the step dimensions; (**c**) and (**d**) variations in the coupling aperture.

To thoroughly assess the impact of varying key design parameters—such as the dimensions of the step introduced on the ridges and the geometry of the coupling aperture—on the performance of the coupler, a study should be conducted. This investigation is essential not only for understanding the sensitivity of the coupler’s electromagnetic behavior to structural modifications, but also for optimizing its functional characteristics, including bandwidth, isolation, and insertion loss. The outcomes of this study play a pivotal role in guiding the selection of appropriate manufacturing techniques and tolerances, ensuring that the fabricated components meet both performance specifications and practical constraints. In CNC-based manufacturing processes, dimensional accuracies on the order of 10 μm are typically achievable and considered standard for precision components. In the present analysis, intentional deviations were introduced in the dimensions of the step and the coupling aperture to simulate fabrication tolerances of ± 50 μm. The corresponding simulation results are illustrated in Fig. 13. As evident from the data, these dimensional variations do not lead to any substantial degradation in the coupler’s performance metrics. Key parameters such as return loss, isolation, and coupling level remain within acceptable bounds, indicating that the design is robust against minor manufacturing imperfections. This finding reinforces the reliability of the proposed structure and confirms its suitability for practical implementation using conventional CNC machining techniques.

## Design of complete structure including transitions

To ensure seamless excitation and integration of the proposed coupler with either an SMA connector or a waveguide flange across the target frequency range, the design must be complemented by the development of ultrawideband transitions. These transitions are essential for enabling efficient energy transfer between the RGW, the coaxial line, and the double-ridge waveguide, thereby enhancing overall performance while maintaining a broad operational bandwidth from 18 to 40 GHz. Here, two transition mechanisms-facilitating seamless integration between RGW and both coaxial and double-ridge waveguide configurations-have been incorporated to ensure efficient excitation across the extensive operational bandwidth.

### Ridge gap waveguide to coaxial line transition

**Fig. 14:**
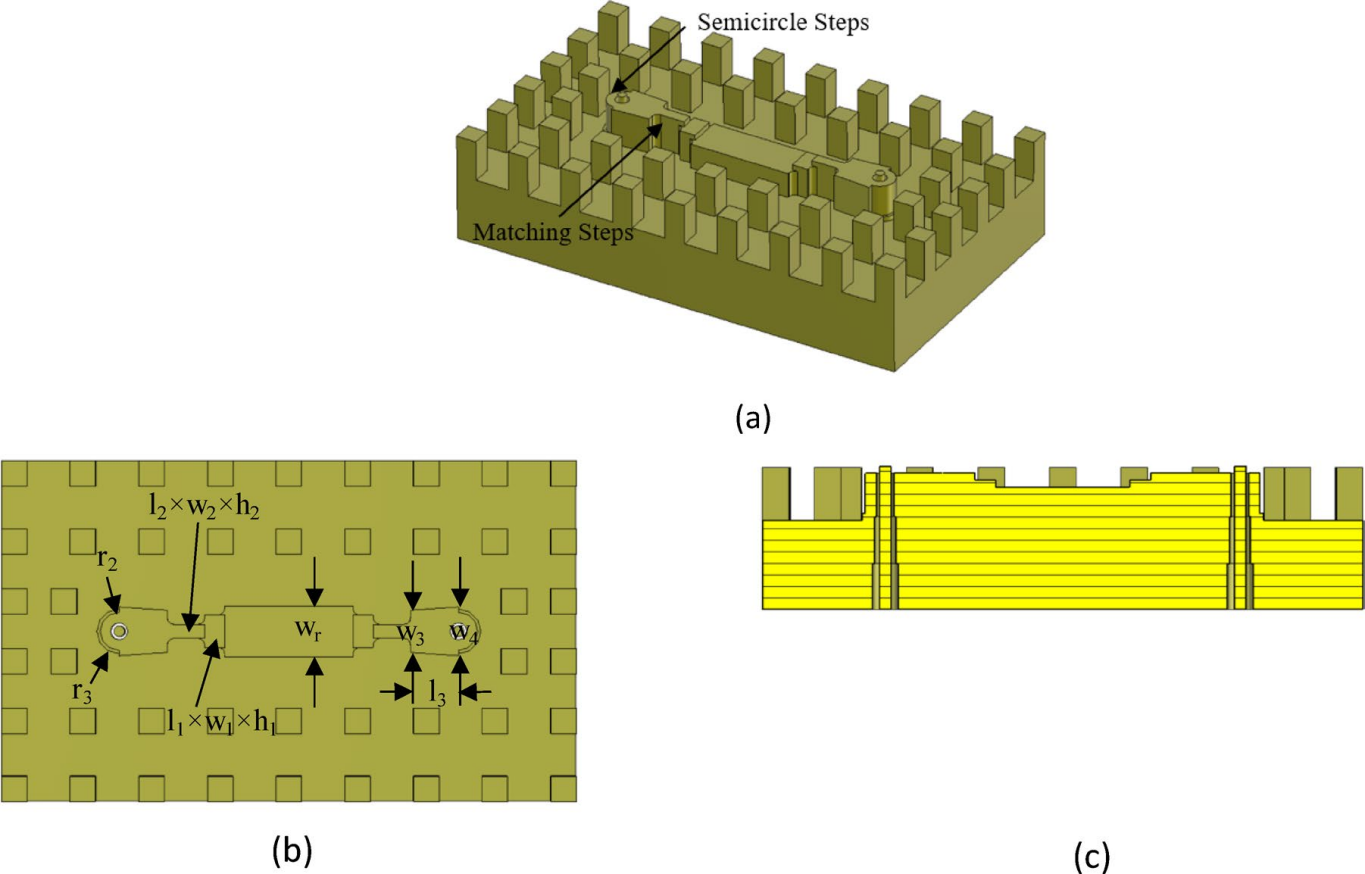
Configuration of back-to-back ridge gap waveguide to coaxial line transitions. (**a**) Perspective view (Top metal plate is not shown). (**b**) Top view. (**c**) Side view.

For the transition design, a back-to-back configuration is employed, where the RGW is positioned between two identical transitions. The target frequency band spans 18 to 40 GHz, with a design goal of achieving a reflection loss better than 20 dB across the entire band. The proposed structure is illustrated in Fig. 14. To realize a wideband transition with excellent impedance matching, several design techniques have been implemented. First, the ridge end is shaped into a semicircle, with a corresponding semicircular step incorporated. Second, the ridge undergoes stepwise variations in both height and width. Third, the 2.92-mm coaxial connector inner conductor traverses a cylindrical hole within the ridge, extending to the opposite metal plate. It is worth noting that the concept of incorporating a semicircular section at the end of the ridge was first introduced in^25^. To extend the bandwidth and fully cover the 18–40 GHz range, an impedance matching technique was also employed by varying both the width and height of the ridge. As shown in Fig. 14c, the radius of this hole also varies in a stepwise manner (*r*_m1_, *r*_m2_). All sharp corners are rounded to a 0.5 mm radius to facilitate machining.

**Fig. 15:**
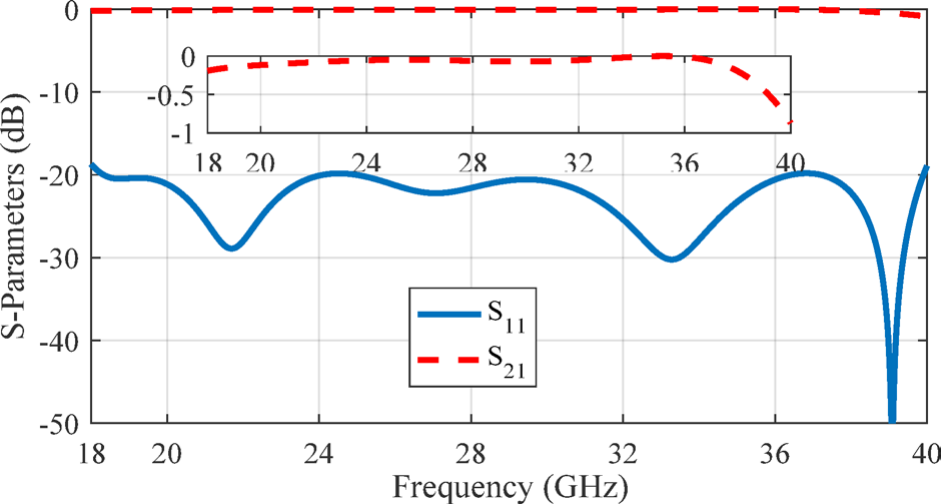
Simulated S-parameters of back-to-back transitions from ridge gap waveguide to coaxial line.

The transition geometry was fine-tuned using CST Microwave Studio and its built-in optimization tools to ensure efficient mode conversion from the RGW to the coaxial TEM mode. The optimized parameters (in mm) are as follows: *l*_1_ = 1.2, *l*_2_ = 2.2, *l*_3_ = 2.8, *w*_1_ = 1.9, *w*_2_ = 0.8, *w*_3_ = 2.4, *w*_4_ = 2.9, *h*_1_ = 2.2, *h*_2_ = 2.7, *r*_2_ = 1.1, *r*_3_ = 1.3, *r*_*m*1_ = 0.6 and *r*_*m*2_ = 0.73 The simulation results for the S-parameters of the optimized structure are depicted in Fig. 15, demonstrating an input reflection coefficient below − 20 dB and a transmission loss of less than 0.5 dB throughout the 18–40 GHz band.

### RGW to double ridge waveguide

**Fig. 16:**
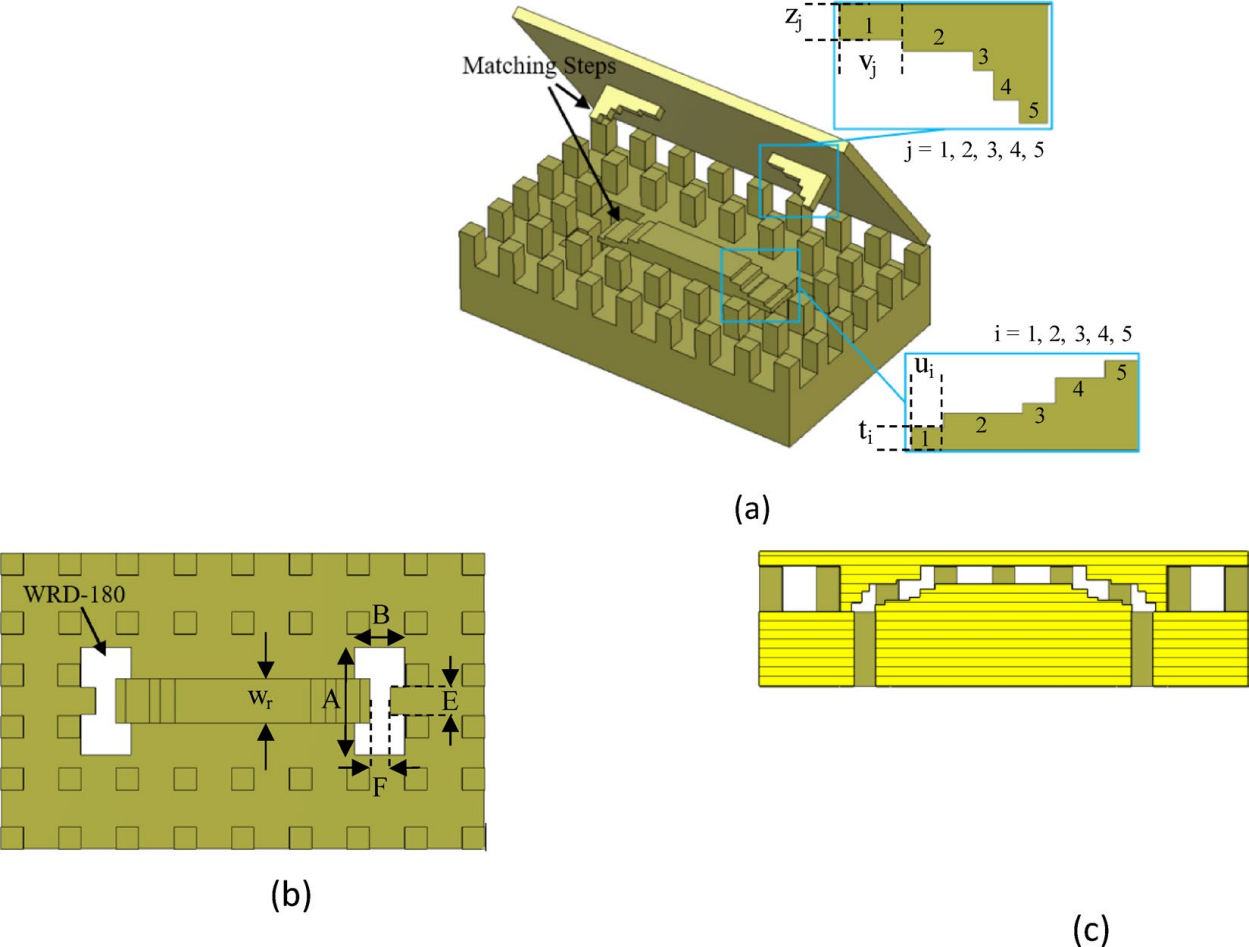
Configuration of back-to-back ridge gap waveguide to double ridge waveguide transitions. (**a**) Perspective view. (**b**) Top view. (**c**) Side view.

As depicted in Fig. 16, the proposed back-to-back structure consists of a RGW, two double ridge waveguide and two mode converter sections. The WRD-180 waveguide has cross-sectional dimensions of *A* = 7.31 mm, *B* = 3.40 mm, *E* = 1.83 mm, and *F* = 1.45 mm, ensuring an extensive single-mode operational bandwidth covering 18 to 40 GHz. The double ridge waveguide does not support TEM mode. Consequently, multiple impedance definitions can be applied to it, as discussed in^41^. Under the power-voltage framework, the characteristic impedance of a double-ridge waveguide is expressed as.

6 $${Z_0}\,=\,\frac{{\pi {\eta _0}F}}{{{\lambda _c}\left( {{C_1}+{C_2}} \right)\sqrt {1 - {{\left( {\frac{{{f_c}}}{f}} \right)}^2}} }}$$

7 $${C_1}=\,\frac{{2F}}{{{\lambda _c}}}{\cos ^2}\left( {\frac{{\pi A}}{{{\lambda _c}}}} \right)\ln \csc \left( {\frac{{\pi F}}{{2B}}} \right)\,+\frac{{\pi E}}{{2{\lambda _c}}}+\frac{1}{4}\sin \left( {\frac{{2\pi E}}{{{\lambda _c}}}} \right)$$

8 $${C_2}\,=\,\frac{F}{B}\frac{{{{\cos }^2}\left( {\frac{{\pi E}}{{{\lambda _c}}}} \right)}}{{\sin \left( {\frac{{\pi (A - E)}}{{{\lambda _c}}}} \right)}}\left( {\frac{{\pi (A - E)}}{{2{\lambda _c}}} - \frac{1}{4}\sin \left( {\frac{{\pi (A - E)}}{{{\lambda _c}}}} \right)} \right)$$

wherein λ_c_ and *f*_*c*_ are the cutoff wavelength and frequency of the dominant mode in the double ridge waveguide, respectively. The cutoff frequency is given by^42^:

9 $${f_c}\,=\,\frac{c}{{2(A - E)}}\left[ {1+\left( {2.45+0.2\frac{E}{A}} \right)\frac{{EB}}{{g(A - E)}}} \right.{\left. {+\frac{{4B}}{{\pi (A - E)}}\left( {1+0.2\sqrt {\frac{B}{{A - E}}} } \right)\ln \csc \left( {\frac{{\pi F}}{{2B}}} \right)} \right]^{ - 1/2}}$$

**Fig. 17:**
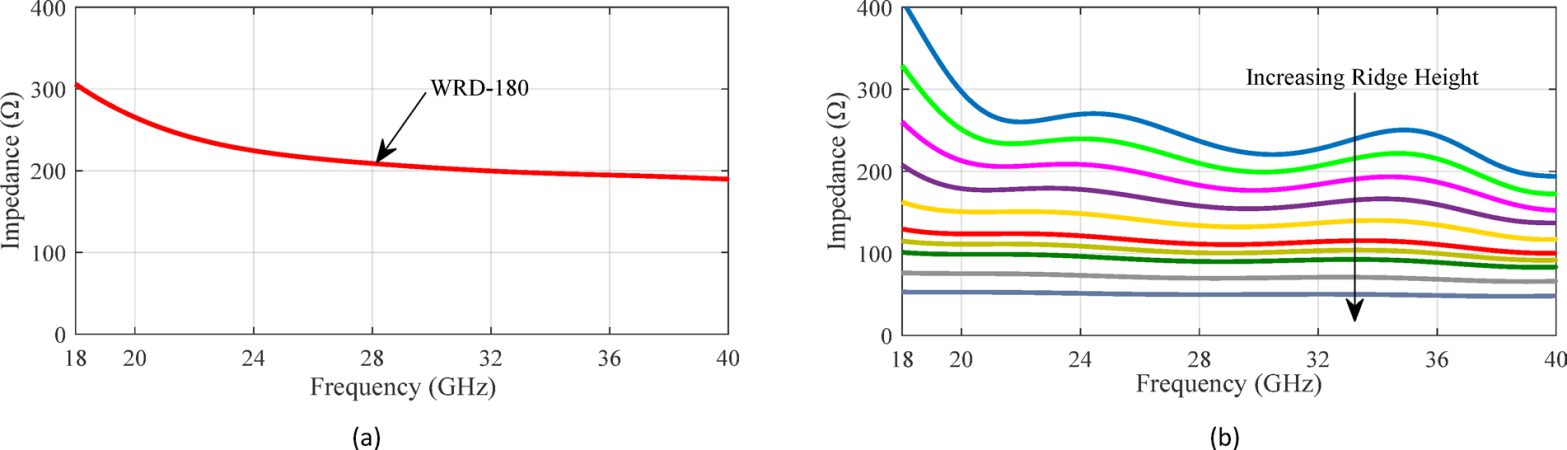
(**a**) Characteristic impedance of WRD-180 double ridge waveguide. (**b**) Variation of impedance of ridge gap waveguide for different values of ridge height.

The characteristic impedance of the WRD-180 waveguide is calculated using Eq. (6) through (9) and is plotted in Fig. 17a. As shown, the impedance varies between 190 Ω and 307 Ω across the desired frequency range. The characteristic impedance of a RGW is approximately given by^10^:

10 $$Z_{0}^{{RGW}}\,=\,\frac{{{\eta _0}}}{{2\left( {\frac{{{W_e}}}{{2{h_r}}}+0.441} \right)}}$$

wherein

11 $$\frac{{{W_e}}}{{2{h_r}}}\,=\frac{W}{{2{h_r}}}\,\left\{ \begin{gathered} 0\,\,\,\,\,\,\,\,\,\,\,\,\,\,\,\,\,\,\,\,\,\,\,\,\,\,\,\,\,\,\,\,\,\,\,\,\frac{W}{{2{h_r}}}\,>\,0.35 \hfill \\ {\left( {0.35 - \frac{W}{{2{h_r}}}} \right)^2}\,\,\,\,\,\,\,\,\,\,\,\frac{W}{{2{h_r}}}\,<\,0.35 \hfill \\ \end{gathered} \right.$$

**Fig. 18:**
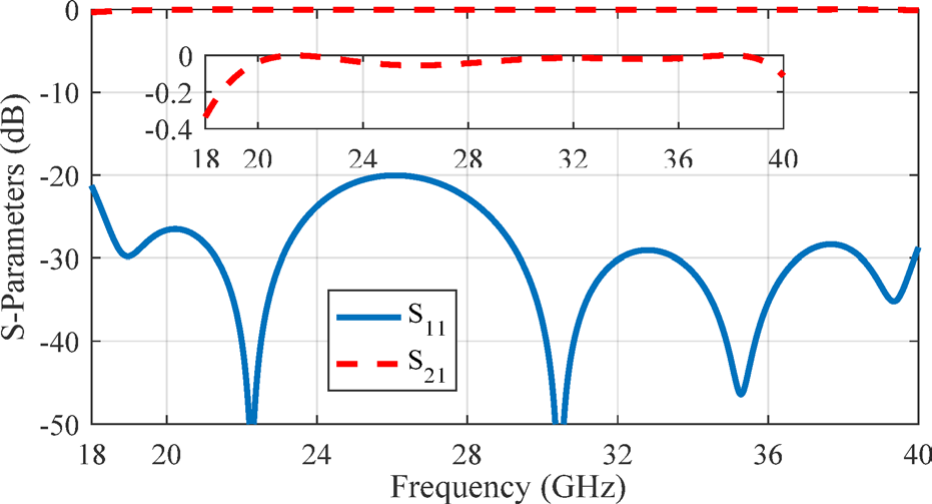
Simulated *S*-parameters of back-to-back transitions from ridge gap waveguide to double ridge waveguide.

This formula offers a quick estimation of the RGW impedance. Its accuracy may be limited, so for more precise and reliable results, full-wave electromagnetic simulation tools such as CST Microwave Studio are preferred. The impedance variation of the RGW with respect to different ridge heights is illustrated in Fig. 17b. The findings indicate that incorporating multi-stepped ridged sections can serve as an effective strategy for facilitating a smooth transition between the RGW and the WRD-180 double ridge waveguide. As shown in Fig. 18, the proposed transition mechanism progressively adapts the RGW into a standard double ridge waveguide through precisely engineered matching steps.

**Fig. 19:**
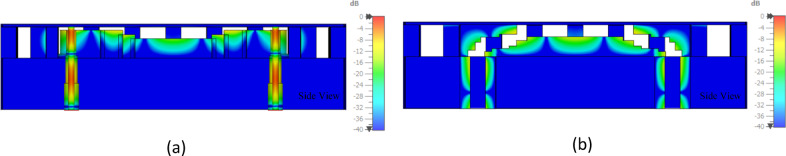
Electric field distribution of transitions at 29 GHz. (**a**) RGW to coaxial line transition. (**b**) RGW to double ridge waveguide transition.

The optimized parameter values of the structure (in mm) are as follows: *t*_1_ = 0.45, *t*_2_ = 0.3, *t*_3_ = 0.2, *t*_4_ = 0.5, *t*_5_ = 0.35, *u*_1_ = 0.65, *u*_2_ = 1.65, *u*_3_ = 0.7, *u*_4_ = 1, *z*_1_ = 0.9, *z*_2_ = 0.3, *z*_3_ = 0.5, *z*_4_ = 0.75, *z*_5_ = 0.6, *v*_1_ = 1.65, *v*_2_ = 1.8, *v*_3_ = 0.5, *v*_4_ = 0.65, *v*_5_ = 0.7. The simulated S-parameter results for the optimized transition, presented in Fig. 18, reveal an input reflection coefficient consistently below -20 dB and a transmission loss maintained under 0.3 dB across the 18–40 GHz frequency range. To demonstrate the performance of two proposed transitions, their electric field distributions at 29 GHz are depicted in Fig. 19.

### Geometry of complete 3-dB coupler

**Fig. 20:**
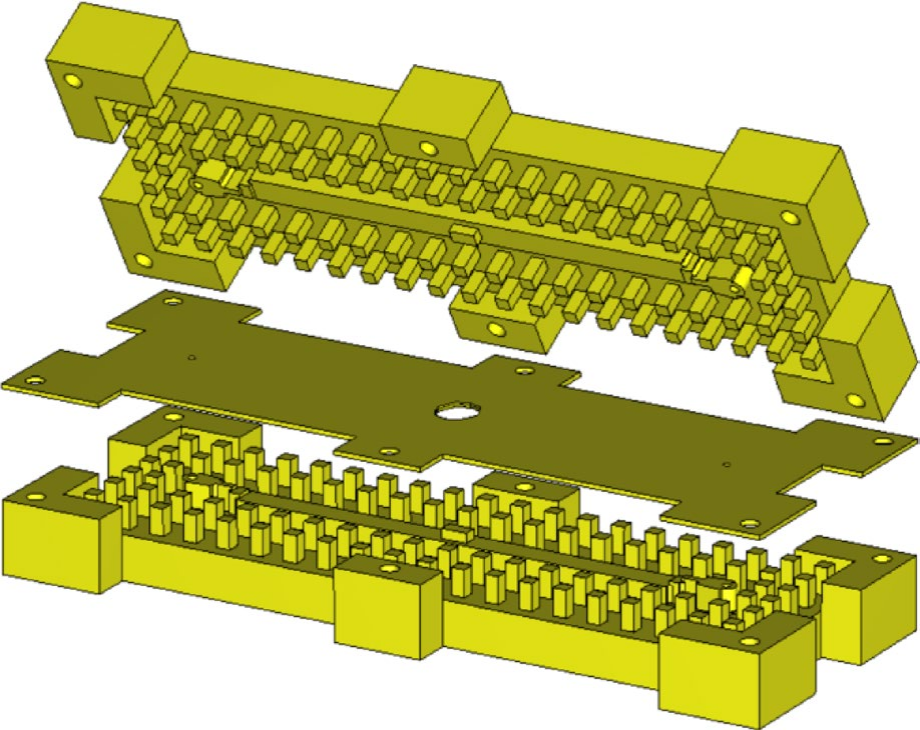
Configuration of complete 3-dB coupler.

**Fig. 21:**
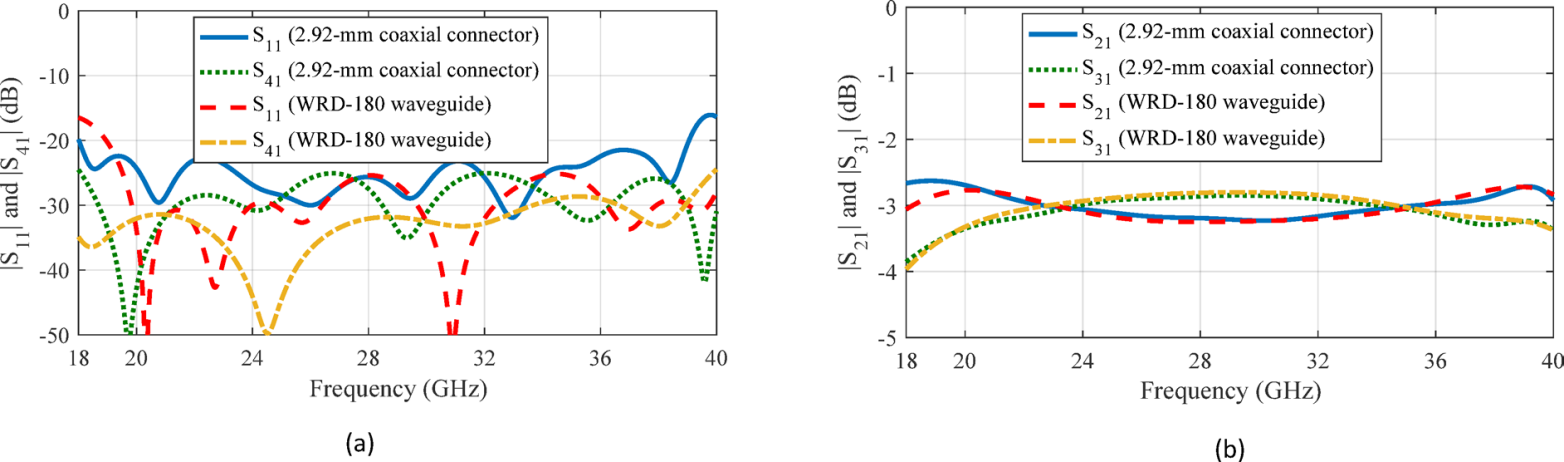
Simulated S-parameters of coaxial- and WRD-180-fed couplers.

The complete design of the proposed coupler, consisting of the coupling region, RGWs, and the transitions from the RGWs to standard 2.92-mm coaxial connector is illustrated in Fig. 20. To achieve a compact overall structure, the transitions are positioned as close as possible to the coupling region. However, this placement leads to multiple wave reflections between these components. To mitigate these effects, the entire configuration is again optimized to achieve the desired input reflection coefficient, isolation, and coupling coefficient within the target bandwidth. The final geometrical parameters for the coaxial- and WRD-180-fed couplers are listed in Table 1 and their simulation results are compared in Fig. 21.

To assess the peak power handling performance of the proposed couplers, the maximum permissible power *P*_*max*_ is calculated by referencing the peak electric field intensity ∣*E*∣_*max*_ within the structure, assuming a normalized input power of 1 W, as governed by:

**Table 1:** Optimized values for design parameters of the coupler.

Parameter	*w*_r_	*w*_1_	*w*_2_	*w*_3_	*w*_4_	*l*_1_	*l*_2_
Value (mm)	3	1.9	0.75	2.35	2.9	1.1	2.1
Parameter	*l*_3_	*h*_1_	*h*_2_	*r*_2_	*r*_3_	*r*_*m*1_	*r*_m2_
Value (mm)	2.8	2.25	2.65	1.1	1.3	0.6	0.73

12 $${P_{\hbox{max} }}\,=\,\,{\left( {\frac{{3 \times {{10}^6}}}{{{{\left| E \right|}_{\hbox{max} }}}}} \right)^2}$$

**Fig. 22:**
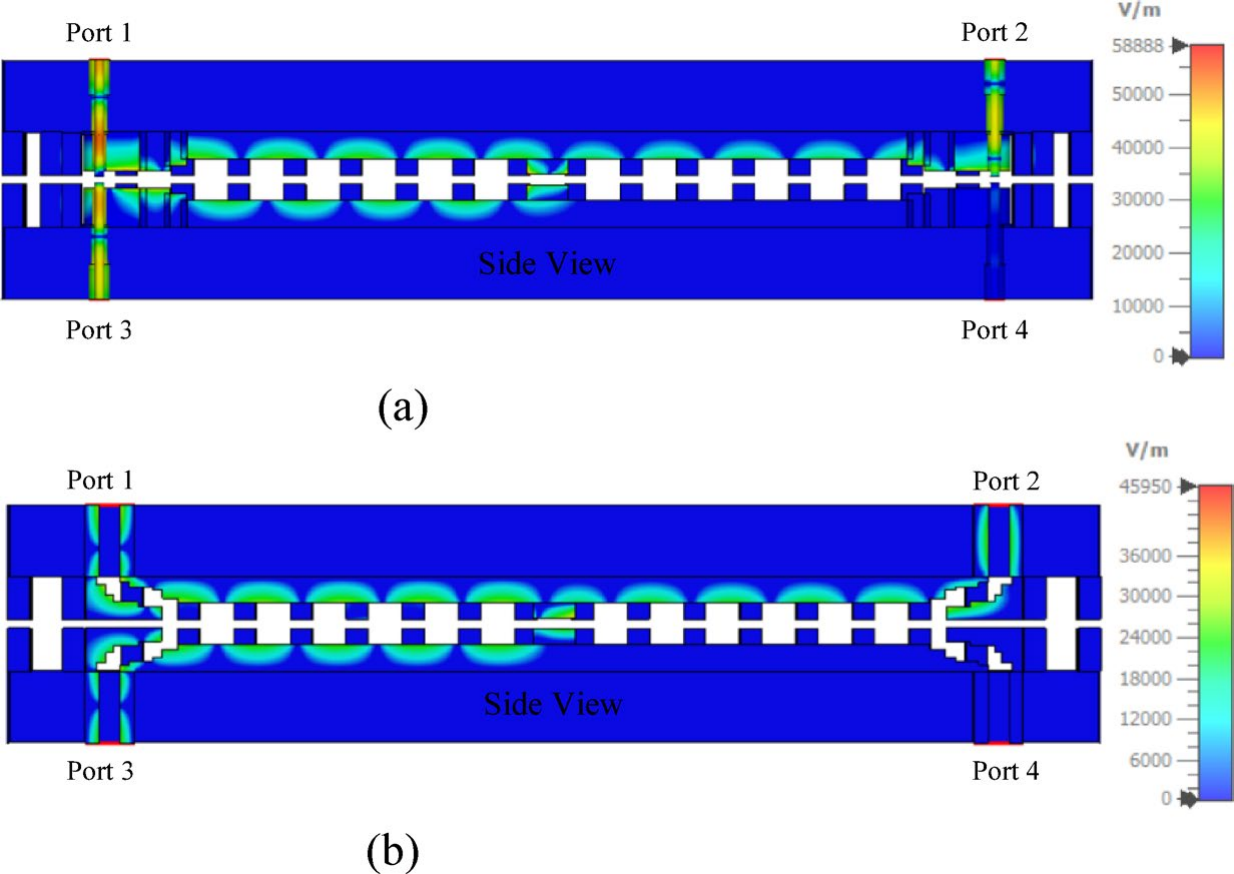
Electric field distribution of two SMA connector- and waveguide-fed couplers at 13 GHz.

As depicted in Fig. 22, at 13 GHz, the SMA connector-fed and waveguide-fed configurations exhibit peak electric field intensities of 58,888 V/m and 45,950 V/m, respectively. These field values correspond to estimated peak power handling capacities of approximately 2.6 kW and 4.3 kW, respectively. Notably, standard 2.92 mm connectors have a maximum power handling capacity of approximately 1 kW, which inherently restricts the overall power capability of the connector-fed coupler.

## Fabrication, measurement, and comparison

### Fabrication

**Fig. 23:**
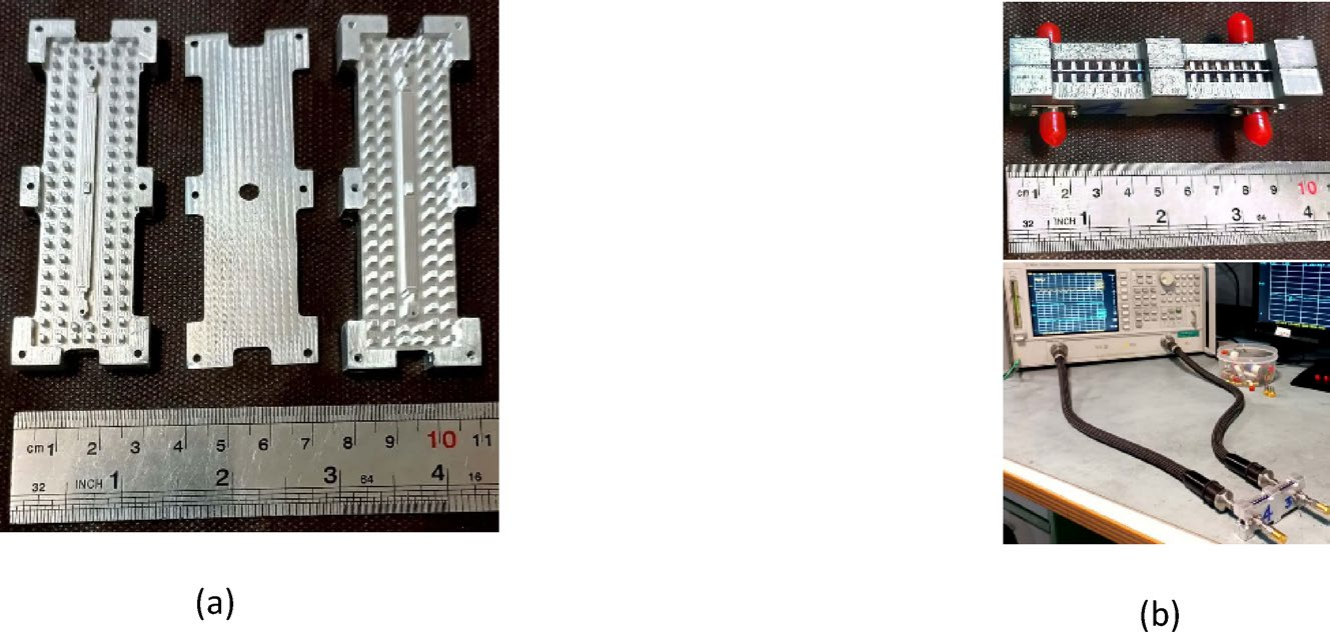
Photographs of (**a**) disassembled and (**b**) assembled fabricated 3-dB coupler.

For the fabrication of a proof-of-concept prototype of the proposed ultrawideband 3-dB coupler, CNC milling was selected due to its precision and accessibility. Aluminum was used as the base material. Since misalignment of structural layers can significantly degrade performance during measurements, alignment pins and screws were incorporated into the design to ensure precise assembly. Images of the fabricated coupler are presented in Fig. 23. The complete prototype, which incorporates support walls for securing the 2.92-mm coaxial connector, has overall dimensions of 85 × 32 × 16 mm³.

### Measurement results

**Fig. 24:**
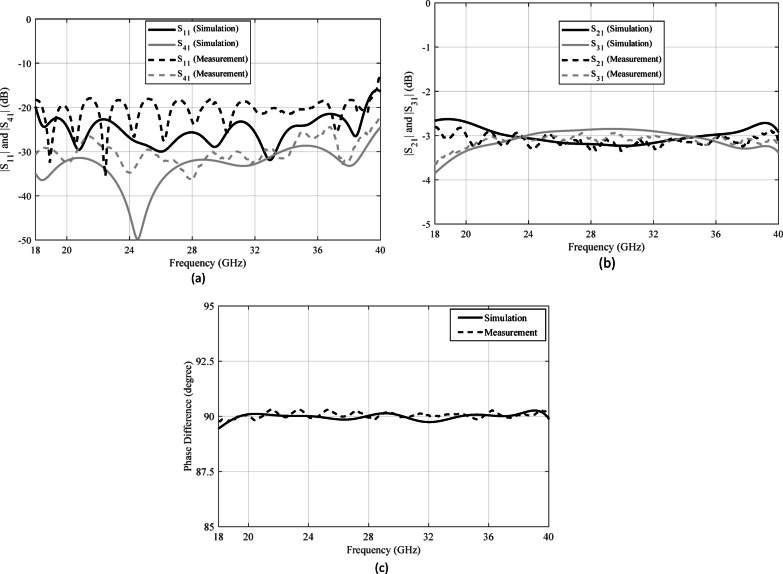
Simulated and measured S-parameters of manufactured wideband gap waveguide-based 3-dB coupler. (**a**) and (**b**) Magnitude of S-parameters. (**c**) Phase difference of through and coupled ports.

The S-parameters are measured using an Agilent 8722ES vector network analyzer. Given the limitations associated with 2.92-mm coaxial connectors, the measurements were conducted within the 18–40 GHz frequency range. As illustrated in Fig. 24, the measurement values are compared with the simulated results of the 3-dB coupler. The findings demonstrate that the fabricated coupler maintained a stable impedance match, with a return loss surpassing 18 dB across a broad bandwidth in the mm-wave frequency spectrum approximately from 18 to 40 GHz. The isolation between waveguides remains above 22 dB, while the coupling levels at the through and coupled ports are measured at 3 ± 0.3 dB with phase imbalance of 1.6° over the same frequency range. Insertion loss is determined by summing the received power at coupled and through ports (in linear scale), subtracting this total from unity to obtain the lost power, and then converting the result into decibels. Simulations indicate an insertion loss of approximately 0.17 dB, while measurements show a slightly higher loss of around 0.51 dB. Although variations in scattering parameters occur at certain frequencies due to connector interfaces and assembly effects, the overall performance remains largely consistent with the simulation data. Small discontinuities and fabrication tolerances at the coaxial-to-RGW transitions and connectors, along with reflections from the measurement setup, appear to introduce unwanted reflections that result in ripple in the return loss.

### Discussion

To assess the effectiveness of the proposed design, Table 2 provides a comparative analysis of various previously reported ridge and groove gap waveguide-based 3-dB couplers alongside the present work. The proposed structure exhibits a wider bandwidth than all the couplers listed in the table, with the exception of^2^. Nevertheless, the coupler in^2^ has lower isolation, and features significantly larger geometric dimensions compared to the proposed design. It is important also to note that while PRGW-based couplers can achieve relatively broad frequency bandwidth and a low-profile form factor, and can be produced using conventional cost-efficient manufacturing processes, they are not well-suited for medium- and high-power applications. In contrast to other studies utilizing metal ridge and groove gap waveguides, the proposed coupler is self-contained, has high power handling capabilities and demonstrates favorable performance across several key parameters, including extremely wide operational bandwidth, compact size, balanced output characteristics, low insertion loss, and consistent coupling behavior. It is worth noting that unlike conventional designs that necessitate an extended coupling region spanning multiple wavelengths (λ_g_) to ensure optimal functionality, the proposed coupler achieves a similar level of performance with a notably reduced coupling region of 0.8 λ_g_. This combination of enhanced performance, compact form factor, and straightforward construction presents a substantial advantage, particularly in mmWave applications, where the fabrication of hollow waveguide-based devices remains a considerable challenge.

**Table 2:** Comparison of the proposed 3-dB coupler with other 3-dB couplers.

Ref	Tech	f_0_ (GHz)	BW (%)	Coupling (dB)	Isolation (dB)	Insertion loss (dB)	Coupling region length (λ_*g*_)
^2^	SIW	10	160	3 ± 0.6	17	0.3	> 6
^9^	HW	2.35	55.3	3 ± 0.34	21.3	N.A	0.7
^13^	PRGW	30	6	3.6 ± 1	12	0.8	1.1
^14^	PRGW	30	13	3.7 ± 0.8	13	1.12	1.12
^15^	PRGW	30	38	3.4 ± 0.5	15	0.55	1.3
^16^	RGW	15.5	14	3 ± 1	15	0.7	1.6
^17^	GGW	14	14.3	3.25 ± 0.75	20	N.A	3
^18^	RGW	18.5	40	3 ± 1	20	0.7	2
^19^	GGW	65.5	25	3 ± 0.5	20	0.65	2
^20^	RGW	11	13	3 ± 1	10	0.35	2
^21^	RGW	16	50	3 ± 0.7	15	N.A	2.2
^22^	GGW	20.5	29.1	3 ± 0.5 dB	15	0.4	1.2
This work	RGW	29	75.8	3 ± 0.3 dB	22	0.51	0.8

## Conclusion

An extremely wideband and compact 3-dB coupler based on gap waveguide technology, incorporating supershaped coupling apertures has been developed, fabricated, and experimentally validated. The challenge of fabrication at high-frequency bands is effectively addressed by utilizing the gap waveguide technology. Additionally, this work applies the superformula to generate complex geometric structures, enabling efficient exploration of an extensive design space with minimal computational effort. To ensure proper excitation and measurement of the proposed coupler across the entire K and Ka band (18–40 GHz), two transition types are introduced: one from RGW to coaxial line and another to a double- ridge rectangular waveguide. Experimental results confirm that the return loss and isolation exceed 18 dB, while the coupler achieves coupling values of 3 ± 0.3 dB at the output ports across the 18–40 GHz range, corresponding to 75.8% fractional bandwidth. The findings indicate that the coupler is highly compatible with standard milling fabrication techniques, and its simple design, broad bandwidth, and compact structure make it a viable option for various mmWave applications.

## Data Availability

All data generated or analysed during this study are included in this published article.
